# Modeling the Effects of Light and Sucrose on *In Vitro* Propagated Plants: A Multiscale System Analysis Using Artificial Intelligence Technology

**DOI:** 10.1371/journal.pone.0085989

**Published:** 2014-01-20

**Authors:** Jorge Gago, Lourdes Martínez-Núñez, Mariana Landín, Jaume Flexas, Pedro P. Gallego

**Affiliations:** 1 Applied Plant and Soil Biology, Faculty of Biology, University of Vigo, Vigo, Spain; 2 Department of Pharmacy and Pharmaceutical Technology, Faculty of Pharmacy, University of Santiago, Santiago de Compostela, Spain; 3 Laboratori de Fisiologia Vegetal, Departament de Biologia, Universitat de les Illes Balears – Instituto Mediterráneo de Estudios Avanzados (UIB-IMEDEA), Palma de Mallorca, Spain; Centrum Wiskunde & Informatica (CWI) & Netherlands Institute for Systems Biology, Netherlands

## Abstract

**Background:**

Plant acclimation is a highly complex process, which cannot be fully understood by analysis at any one specific level (i.e. subcellular, cellular or whole plant scale). Various soft-computing techniques, such as neural networks or fuzzy logic, were designed to analyze complex multivariate data sets and might be used to model large such multiscale data sets in plant biology.

**Methodology and Principal Findings:**

In this study we assessed the effectiveness of applying neuro-fuzzy logic to modeling the effects of light intensities and sucrose content/concentration in the *in vitro* culture of kiwifruit on plant acclimation, by modeling multivariate data from 14 parameters at different biological scales of organization. The model provides insights through application of 14 sets of straightforward rules and indicates that plants with lower stomatal aperture areas and higher photoinhibition and photoprotective status score best for acclimation. The model suggests the best condition for obtaining higher quality acclimatized plantlets is the combination of 2.3% sucrose and photonflux of 122–130 µmol m^−2^ s^−1^.

**Conclusions:**

Our results demonstrate that artificial intelligence models are not only successful in identifying complex non-linear interactions among variables, by integrating large-scale data sets from different levels of biological organization in a holistic plant systems-biology approach, but can also be used successfully for inferring new results without further experimental work.

## Introduction

Since the beginning of *in vitro* culture in 1902 when the Austrian botanist Gottlieb Haberlandt attempted to grow isolated plant cells and tissues (leaf mesophyll and hair cells) in nutritive solutions, a large body of work has emerged describing the optimization of different culture conditions to supply explants with all the components required for successful *in vitro* plant tissue propagation. During the past 70–80 years, more than 3000 scientific articles have described the use of over 2000 different culture media in plant tissue culture [1]. *In vitro* tissue propagation, however, is still a stressful procedure for plants, which can limit the successful establishment of plants upon transfer to *ex vitro* conditions [2]–[5]. In many cases, the best *in vitro* conditions do not lead to optimal *ex vitro* results. Therefore, a better understanding of the complex effects of the variables involved during the *in vitro* plant tissue growth on the *in vitro* culture and the *ex vitro* acclimatization results should lead to an improvement of the process. The effect of carbon in the media, light conditions and their interaction appear to be particularly important [6]–[8].

Sucrose is the most common carbon source used in plant cell, tissue and organ culture. Media with 3% sucrose have been the staple since Murashige and Skoog [9] described their MS medium. Sucrose acts during plant tissue culture as a fuel source for sustaining photomixotrophic metabolism, ensuring optimal development, although other important roles such as carbon precursor or signaling metabolite have more recently been highlighted [10]–[13]. Sucrose also supports the maintenance of osmotic potential and the conservation of water in cells. However, high sucrose concentration in the media restricts the photosynthetic efficiency of cultured plants by reducing the levels of chlorophyll, key enzymes for photosynthesis and epicuticular waxes promoting the formation of structurally and physiologically abnormal stomata [3]. On the other hand, earlier studies have shown that plantlets growing under tissue culture conditions do not fix enough CO_2_ to sustain growth in the absence of sucrose, which is mainly due to limited CO_2_ inside the vessel [14]–[18].

High irradiance and low air humidity, during the subsequent acclimation phase are also stressful to plantlets when they are just starting to become photoautotrophic [19]–[21]. These limitations of *in vitro*-developed plants, many of which are specifically related to a low photosynthetic efficiency and a low capacity of regulating water loss, prompted the design of a large number of micropropagation protocols trying to favor the development of high photosynthesis capacity and subsequent *ex vitro* acclimatization [2], [22]–[30]. Most of these studies focused on discovering and identifying the best parameter(s) for an easy and fast assessment of the quality of *in vitro* cultured plantlets with regards to acclimation. Physiological parameters at subcellular levels, such as chlorophyll fluorescence, were widely proposed as a useful indicator of plant quality of acclimated plants [11], [31]–[33]. However, the use of chlorophyll fluorescence to assess the photoinhibition caused by the transfer of *in vitro* plants *to ex vitro* conditions has produced controversial results: while some researchers [34]–[38] found the largest photoinhibition in the least photoautotrophic rose plantlets; others [29] described that gardenia plantlets cultured under conventional sucrose concentration and irradiance, indeed photomyxotrophic plantlets, were the least photoinhibited. It seems clear, that a single level of response (any from subcellular up to whole plant scale) does not determine the quality of the plant due to the complexity of the responses of plants to the factors and their interactions at different levels of biological organization [39]. For instance, *in vivo* chlorophyll fluorescence cannot correlate with plant photosynthesis rate due to stomatal limitations [40] or the leaf level photosynthesis may not necessarily correlate with plant growth [41]–[42]. Hence, for proper development of an *in vitro* culture protocol, consideration should be given to analyzing the effect of *in vitro* factors (as sucrose or light) on parameters at the different levels of organization in a holistic plant system-biology approach. A review of the literature indicates that the evaluation of *in vitro* factor effects on the quality parameters of plants are typically performed using conventional statistical analysis of variance together with multiple comparison tests [43].

The development of platforms to integrate multidimensional and multiscale data and to derive models for explaining the process as a whole remains one of the main goals for the plant scientific community [45]–[49]. Soft-computing techniques, such as Artificial Neural Networks (ANN), appear to be quite promising in addressing complex analyses in biological studies [50]. ANNs are mathematical tools useful for modeling non-linear relationships between variables. Compared to conventional statistics, ANN has shown higher accuracy in prediction as pointed out in several plant science papers [43]–[44], [51]–[53] as well as in other scientific areas such as pharmaceuticals [54]–[55]. Recently, we have used a combination of ANN and fuzzy logic technology (neurofuzzy logic) to model complex multivariate datasets in order to find the best combination of factors for *in vitro* culture of grapevine [56] or to extract knowledge on apricot *in vitro* culture conditions from an historical collection of data via data mining [57]. However, these previous analyses were carried out using data from a single level of biological organization (one scale model). To the best of our knowledge, the utility of artificial intelligence to perform an analysis of the effect of *in vitro* factors on several parameters at different levels of biological organization (a multiscale approach) has never been proposed.

The advantage of the neurofuzzy logic technology for this purpose lies in its ability to process and model information and to present results in the form of linguistic terms (IF-THEN rules) and membership degrees [50]. Linguistic terms are the human tools to solve problems, make decisions or draw conclusions [50 and references therein].

In the present study, we test the validity of neurofuzzy logic as an appropriate strategy for modeling multivariate data and its effects on multiscale parameters for a better understanding and an improvement in the plant acclimation process. Specifically, the objectives of this work were: to assess the effectiveness of neurofuzzy logic technology in modeling multiscale data sets; to discover hidden knowledge and retrieve new insights into the regulation of sucrose and light on *in vitro* kiwifruit plant acclimation and, finally, to infer the optimal combination of plant traits to achieve the best acclimation.

## Materials and Methods

### Plant Material and *in vitro* Culture Conditions

The experiments were carried out using micro-shoots of kiwifruit *Actinidia deliciosa* (A. Chev) C. F. Liang et A. R. Ferguson var. *deliciosa* cv. Hayward as described elsewhere [43]. Briefly, micro-shoots were proliferated in Cheng medium [58] containing 1 mg L^−1^ BAP (6-benzylaminopurine), 1 mg L^−1^ GA_3_ (gibberellic acid), sucrose (at 6 different concentrations; see below) and 0.8% w/v Plant Agar (Duchefa®). Media pH was set to 5.7 prior autoclaving (121°C, 1 kg cm^−2^ s^−1^ for 15 min). The cultures were maintained under a 16 h-photoperiod at three different light intensities (see below) and at temperatures of 25±2°C during the day and 22±2°C at night, during two subcultures of 28d. The experiments followed a factorial design for two variables (inputs): sucrose concentration at 6 levels (0, 1.0, 1.5, 2.0, 2.5 and 3.0% w/v) and light treatment PPFD (Photosynthetic Photon Flux Density) at 3 levels: low light (LL, 60 µmol m^−2^ s^−1^), medium light (ML, 100 µmol m^−2^ s^−1^) and high light (HL, 200 µmol m^−2^ s^−1^). Each light and sucrose treatment consisted of five replicates of three explants each. Every experiment was repeated at least threefold.

### *Ex vitro* Simultaneous Rooting and Acclimatization Culture Conditions

Micro-shoots longer than 1.5 cm, after removal from *in vitro* proliferation cultures, were quick-dipped (1 min) at their basal side, into a filter-sterilized auxin solution of 25 mM IAA (indole-3-acetic acid). They were carefully planted into mini-pots containing planting mixture (perlite: compost 1∶1), covered with plastic tubes and placed in a growth room (Sanyo model SGC066.CFX.F) under a 16h-photoperiod. The light was provided by fluorescent lamps (Philips TLD32W/83HF) with light intensity of 80±10 µmol m^−2^ s^−1^ at the level of the ground. Temperature was 25±2°C during the day and 20±2°C at night. The initial value of RH (relative humidity) was set to 100% and decreased gradually over 45 days to 70%. Plantlets were watered daily.

### Data Acquisition

At the end of the *ex vitro* phase allowing simultaneous rooting and acclimatization, plantlets were harvested and a total of 14 parameters (outputs) grouped at 3 different levels were recorded. Parameters were distributed into three biological organization scales, as proposed by Lucas and coworkers [59]: whole-plant (8 parameters), tissue (2 parameters) and subcellular level (4 parameters):

1.– Whole plant scale. After 45 days of *ex vitro* simultaneous rooting and acclimatization, eight parameters (outputs) were recorded to analyze the effects of the variables (inputs) on growth: 1) survival percentage; 2) root length of the longest root measured from the basis of the shoot to the root apex (cm); 3) shoot length measured from the basis of the shoot to the shoot apex (cm); 4) number of *in vitro* leaves per plantlet (leaves formed during *in vitro* stage); 5) number of *ex vitro* leaves per plantlet (leaves formed under *ex vitro* conditions); 6) *ex vitro/in vitro* leaves index (ratio of the leaves formed under *ex vitro* and *in vitro* conditions); 7) plantlet dry weight (60°C until constant weight) and 8) plantlet water content (WC) percentage calculated as follows:

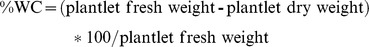



Leaves formed under *in vitro* conditions are distinguished from those formed under *ex vitro* conditions. Leaves originated under *in vitro* tissue conditions inside the culture vessel (highly controlled environment, low light and external sugar addition) are typically described using several morpho-anatomical, histological (reduced epicuticular waxes and/or abnormal no functional stomata along the leaf) and physiological (low levels of chlorophylls, key enzymes for photosynthesis promoting a restricted photosynthetic efficiency considering them in many cases as reservoirs) features, in contrast to leaves formed outside the culture vessel under *ex vitro* tissue culture conditions whose traits approximate the typical traits of the species [3], [29].

2.– Tissue scale. Leaf stomatal characteristics in kiwifruit were studied following the methodology proposed by Moncaleán et al. [60] i.e. the second or third apical fully expanded leaves from plantlets after 45 d of simultaneous *ex vitro* rooting and acclimatization, were collected and fixed 16h in ethanol 70% for scanning electron microscopy (SEM). Fixed leaves were further dehydrated, by increasing the ethanol solution concentrations from 70% to 100% (v/v). Dehydrated samples were placed into iso-amyl-acetate solution and dried at 37°C at a pressure of 1200–1500 psi in a CO_2_ atmosphere using a critical point CPD030 (Bal-Tec) dryer. Metallization of the explants by cathodic deposition with gold-paladium in argon atmosphere (1 min, t 20 mÅ, 2.2 KW) (Emitech K550X) was performed on aluminum stubs. Abaxial leaf surfaces of three leaves per treatment and 12 randomly chosen visual fields (20 µm^2^) per leaf were viewed (at 600x) in a computer-controlled (Phillips XL 30) SEM. Two parameters were recorded: stomatal density (number of total stomata per mm^2^) and percentage of open stomata.
3.– Subcellular scale. Chlorophyll fluorescence parameters were obtained from the last fully developed leaves of 12–20 plantlets after 45d of *ex vitro* simultaneous rooting and acclimatization. A pulse-amplitude modulation system fluorometer (PAM-2100, Heinz Walz Gmbh) was used to measure modulated fluorescence following the methodology described by Carvalho *et al*. [31]. *In vivo* chlorophyll fluorescence emission from the upper leaf surface was measured on dark adapted leaves (30 min). Two fluorescence parameters were measured: ground fluorescence F_0_ and maximal fluorescence F_m_ using light of <0.1 µmol m^−2^ s^−1^ intensity and after a saturated pulse of >3500 µmol m^−2^ s^−1^ intensity, respectively. The maximal variable fluorescence (F_v_ = F_m_−F_0_) and the potential quantum efficiency of PSII (F_v_/F_m_) were calculated [61]. F_0_ and F_v_/F_m_ were modeled to determine the inhibition of PSII.

The photosynthetic pigments were determined from fully expanded second or third apical leaves of each plantlet collected at 45 d of simultaneous *ex vitro* rooting and acclimatization. Pigments were determined after homogenizing and macerating the samples in acetone at room temperature. Two parameters: Chlorophyll (a and b) and total carotenoid (carotene and xanthophyll) concentration were determined spectrophotometrically following method of Lichtenthaler [62]. For each light treatment and sucrose concentration 12–20 samples were analyzed. Results are expressed in µg g^−1^ of fresh leaf weight.

### Neurofuzzy Logic

A neurofuzzy logic approach to modeling *in vitro* plant acclimation of kiwifruit plantlets was implemented. Neurofuzzy logic is a hybrid approach that combines the strength and the adaptive learning capabilities of neural networks with the ability to generalize rules from fuzzy logic. Specifically, ASMOD (Adaptative Spline Modeling of Observation Data) has been employed [63]. This method uses global partitioning that involves splitting the model into smaller submodels. Various models and submodels were examined, starting from a set of the simplest models. The models are sums or products of the basic functions, producing submodels that depend only on a subset of the inputs [50], [56]. In this study, we used the FormRules v3.31 software (Intelligensys Ltd, UK) to develop a neurofuzzy logic multiscale model.

The neurofuzzy logic application finds a predictive model for each parameter measured, named here as output, and generates a set of “IF-THEN” rules with different values of membership degree [50]. Complex models are simplified to make them as simple as possible and to perform under easily understandable rules.

This neurofuzzy logic application contains various statistical fitness criteria with the best results found when Structural Risk Minimization (SRM) was used. The training process [50] was conducted as reported by Shao and coworkers [54]. Minimization parameters are summarized in Table 1.

**Table 1:** The training parameters setting with FormRules v3.31.

**Minimization parameters**	
Ridge Regression Factor: 1 e^−6^	
**Model Selection Criteria**	
Structural Risk Minimization (SRM)	
C1 = 0.530–0.836	C2 = 4.8
Number of Set Densities: 2	
Set Densities: 2, 3	
Adapt Nodes: TRUE	
Max. Inputs Per SubModel: 4	
Max. Nodes Per Input: 15	

The accuracy of the neurofuzzy logic model was further evaluated using the correlation coefficient (R^2^) for each output.
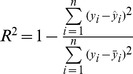
Where 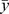 is the mean of the dependent variable, and ŷ is the predicted value from the model. The larger the value of the Training Set R^2^, the more the model captured the variation in the training data. Values between 0.70–0.99 are indicative of reasonable model accuracy [64]. Values of ANOVA *f*-test statistic higher than upper critical values of the *f* distribution for the degrees of freedom used for each parameter indicate no significant differences between experimental and predicted data (α<0.05) and, therefore, high model predictabilities.

## Results

### Model Predictability

Neurofuzzy logic submodels were successfully and simultaneously developed for the 14 parameters (outputs) as a function of two variables (inputs): sucrose concentration and light intensity (Table 2). The number of submodels, the significant inputs and their interactions, the correlation coefficients and ANOVA results for each parameter are shown in Table 3. Correlation coefficients for all the parameters are over 0.71 indicating reasonable accuracy of our model. The neurofuzzy logic approach succeeded in identifying significant single as well as interactive effects of variables on parameters measured.

**Table 2:** Dataset with the *in vitro* culture conditions (inputs) and *ex vitro* acclimatization parameters (outputs) modeled by neurofuzzy logic.

INPUTS		OUTPUTS													
		Whole plant level								Tissue level		Subcellular level			
PPFD (μmolm^−2^ s^−1^)	Sucrose (%)	Survival (%)	Rootlength(cm)	Shootlength(cm)	*In vitro*leavesperplantlet	*Ex vitro*leavesperplantlet	*Ex vitro/* *in vitro*leaves	Plantletdryweight(g)	WC (%)	Stomataldensity(mm^−2^)	Openstomata(%)	F_v_/F_m_	F_0_	Chl a+b(μg g^−1^ leaf)	Carotenoids(μg g^−1^ leaf)
200	0.0	53.6±0.7	2.0±0.4	2.6±0.1	4.1±0.1	2.0±0.0	0.5±0.0	1.1±0.0	34.0±0.2	480.9±38.7	80.58±5.7	0.709±0.01	0.092±0.004	546.3±27.7	105.4±9.0
200	1.0	84.4±2.1	2.9±1.2	3.3±0.3	3.7±0.2	1.8±0.1	0.8±0.1	1.0±0.1	44.3±0.3	555.6±20.2	65.15±7.4	0.727±0.01	0.092±0.004	792.1±37.3	191.2±13.6
200	1.5	95.6±1.1	4.1±3.7	3.7±0.2	2.7±0.1	2.0±0.0	0.9±0.0	1.1±0.1	45.4±0.2	637.5±17.1	68.45±4.4	0.733±0.00	0.089±0.002	694.9±73.5	126.0±18.2
200	2.0	100.0±0.0	3.0±1.4	4.2±0.6	2.3±0.2	2.6±0.1	1.2±0.0	1.2±0.0	43.1±0.1	572.2±26.6	100.04±2.3	0.743±0.01	0.090±0.003	734.3±60.8	156.3±12.1
200	2.5	91.6±1.0	5.4±1.4	5.0±0.7	2.9±0.3	2.8±0.1	1.0±0.1	1.2±0.1	53.5±0.1	729.2±21.6	84.57±1.7	0.734±0.00	0.084±0.002	811.5±62.5	168.9±15.2
200	3.0	100.0±0.0	5.3±1.5	5.1±0.7	2.3±0.2	2.9±0.1	1.3±0.0	1.2±0.1	58.4±0.1	251.9±5.7	95.41±2.4	0.735±0.00	0.088±0.002	1586.7±70.6	251.5±16.7
100	0.0	61.3±1.7	4.6±1.2	3.6±0.1	3.0±0.4	2.3±0.0	0.8±0.0	1.1±0.0	42.0±0.1	879.2±29.9	18.96±1.7	0.708±0.01	0.085±0.003	711.9±38.8	135.7±4.5
100	1.0	80.0±1.4	5.0±1.2	4.2±0.1	3.3±0.3	2.0±0.0	0.6±0.1	1.2±0.1	50.6±0.0	645.8±19.4	35.29±1.8	0.719±0.01	0.086±0.003	942.4±58.0	165.4±10.6
100	1.5	90.0±0.7	6.7±2.4	5.5±0.4	2.9±0.1	2.9±0.2	1.0±0.1	1.3±0.1	58.8±0.0	406.3±31.1	31.28±6.1	0.730±0.01	0.088±0.003	863.8±76.0	159.8±8.6
100	2.0	91.1±1.1	6.4±0.7	5.0±0.3	3.0±0.1	2.4±0.1	0.8±0.1	1.3±0.1	56.8±0.1	333.3±13.7	52.50±9.1	0.717±0.01	0.094±0.002	1371.6±45.6	246.9±8.4
100	2.5	87.1±1.4	8.1±1.2	6.2±0.3	3.0±0.1	3.2±0.1	1.1±0.1	1.3±0.1	65.0±0.1	470.8±36.9	39.82±5.0	0.733±0.01	0.092±0.002	1371.6±81.3	246.9±11.2
100	3.0	91.5±0.7	7.3±1.6	5.4±0.1	2.5±0.2	2.8±0.1	1.1±0.2	1.2±0.1	58.3±0.0	533.3±30.3	34.38±3.2	0.716±0.01	0.093±0.002	1379.1±86.5	227.9±9.0
60	0.0	51.9±2.3	3.7±2.9	3.4±0.3	3.2±0.3	2.6±0.1	0.9±0.1	1.1±0.1	43.3±0.1	314.6±2.3	39.07±1.1	0.731±0.02	0.050±0.002	1082.1±114.6	196.9±23.5
60	1.0	88.9±1.5	4.9±2.6	4.6±0.1	2.9±0.2	3.0±0.1	1.1±0.1	1.1±0.1	50.4±0.2	341.7±25.8	69.51±4.7	0.747±0.01	0.045±0.002	1277.2±41.8	212.1±7.6
60	1.5	81.7±2.5	5.1±3.1	4.8±0.1	3.2±0.1	2.9±0.1	1.1±0.1	1.2±0.1	49.8±0.2	762.5±40.5	34.66±3.4	0.731±0.01	0.049±0.002	1269.3±72.1	284.4±24.3
60	2.0	93.3±2.2	4.8±1.2	4.7±0.1	2.9±0.1	2.8±0.1	1.0±0.1	1.2±0.1	47.9±0.0	862.4±39.6	-	0.738±0.01	0.095±0.002	1278.7±38.1	211.6±6.9
60	2.5	90.5±2.5	6.3±0.7	4.9±0.3	2.8±0.1	3.0±0.2	1.1±0.2	1.2±0.2	53.0±0.1	937.5±39.4	29.02±1.1	0.746±0.01	0.086±0.001	1301.1±39.1	198.5±23.0
60	3.0	88.9±1.1	7.2±1.5	5.3±0.2	3.0±0.1	3.4±0.1	1.1±0.1	1.2±0.1	59.0±0.0	900.0±29.7	23.61±1.8	0.741±0.01	0.081±0.001	1039.8±45.5	179.5±8.3

Data shows the mean of 12–30 samples ± SE (see material & methods).

**Table 3:** Significant inputs from neurofuzzy logic submodels and training R^2^ with *f* value, degrees of freedom and p-value (99 and 95%) in the ANOVA for each output.

Outputs	Submodel	Significant inputs and interactions	R^2^	*f* value	df1,df2*	*α* value
Survival (%)	1	S	0.8771	33.31	3, 17	<0.01
Root length (cm)	1	**S**	0.8938	27.36	4, 17	<0.01
	2	L				
Shoot length (cm)	1	**S**	0.8628	20.44	4, 17	<0.01
	2	L				
*In vitro* leaves per plantlet	1	S×L	0.8239	5.26	8, 17	<0.01
*Ex vitro* leaves per plantlet	1	**S**	0.7275	6.41	5, 17	<0.01
	2	L				
*Ex vitro/in vitro* leaves	1	S×L	0.7488	5.47	6, 17	<0.01
Plantlet dry weight (g)	1	S×L	0.9550	2.83	15, 17	<0.05
WC (%)	1	**S**	0.8210	14.91	4, 17	<0.01
	2	L				
Stomatal density (mm^−2^)	1	S×L	0.9493	2.50	15, 17	<0.05
Open stomata (%)	1	L	0.7564	14.68	3, 16	<0.01
F_v_/F_m_	1	**L**	0.7149	6.02	5, 17	<0.01
	2	S				
F_0_	1	S×L	0.7555	5.66	6, 17	<0.01
Chl a+b (µg g^−1^ leaf)	1	S×L	0.9825	7.46	15, 17	<0.01
Carotenoids (µg g^−1^ leaf)	1	S×L	0.9787	6.13	15,17	<0.01

Inputs: S, sucrose and L, light. Inputs with the stronger effect on each output are highlighted.

df: degrees of freedom; df1: model; df2: total.

When two inputs have independent effects on an output the most important effect is pointed out as submodel 1 (Table 3). For example, sucrose has an independent and stronger effect than light intensity on root length, while light intensity has a stronger effect than sucrose on F_v_/F_m_. A unique submodel (labeled 1) is identified if only one input has an effect over an output. As an example, the sucrose concentration determines the survival of plantlets during acclimation whatever the light intensity, however, light intensity determines the percentage of open stomata in leaves regardless the amount of sucrose concentrations (Table 3). Additional significant independent effects of either input (where no interactions between them were observed) can be seen on the following outputs: root and shoot length, *ex vitro* leaves, WC (%) and F_v_/F_m_. Finally, a significant interaction between sucrose and light was found for the following outputs: number of *in vitro* leaves, *ex vitro*/*in vitro* leaves, plantlet dry weight, stomatal density, F_0_, chlorophyll a+b and carotenoids. In summary, using a neurofuzzy logic approach an accurate model was produced that provides, in an easy way, clear and precise information on the effect and interaction of both variables studied upon 14 parameters.

### Whole Plant Scale

**Figure 1:**
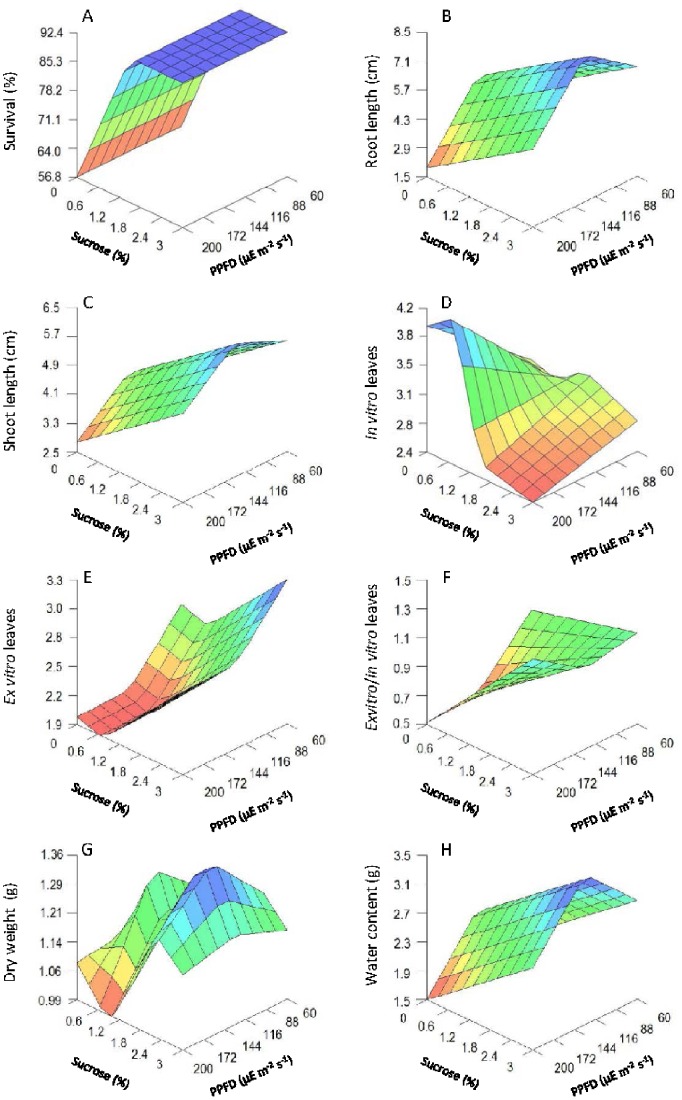
3D plots of growth parameters predicted by the neurofuzzy logic model for kiwifruit plantlets at 45 d at acclimatization stage as a function of sucrose added in the medium and light intensities used during *in vitro* culture. (A) Survival (%). (B) Root length (cm). (C) Shoot length (cm). (D) Number of *in vitro* leaves per plantlet. (E) Number of *ex vitro* leaves per plantlet. (F) *Ex vitro/in vitro* leaves. (G) Plantlet dry weight. (H) Plantlet water content (%).

Growth parameter data predicted by the model as a function of both the sucrose percentage and the light intensity are presented in 3-D plots (Fig. 1).

As mentioned above, only sucrose had a positive effect on the survival parameter (Table 3), but it is interesting to note that the survival percentages were all over 90% when a minimum sucrose concentration was used (c.a. 1.2%) regardless of the light intensity (Fig. 1A).

Root length (Fig. 1B), shoot length (Fig. 1C) and WC (Fig. 1H) showed a significant independent effect on both variables following a similar qualitative pattern. The highest values were achieved with high sucrose concentrations (c.a. 3%) and medium light levels (in the range of 122–138 µmol m^−2^ s^−1^). Increasing the sucrose concentration promoted an increase in these parameters, especially in the length of the shoots, but suboptimal and supraoptimal light intensities clearly inhibited the growth of these organs and the whole organism (measured as dry weight, Fig. 1G).

With reference to leaf development, opposite trends were observed when comparing *in vitro* (Fig. 1D) and *ex vitro* leaves (Fig. 1E). The lowest sucrose concentration and the highest light intensity yielded the highest number of *in vitro* leaves whilst the highest number of *ex vitro* leaves per plantlet (3.32) was achieved with typical *in vitro* culture conditions consisting of low light (c.a. 60 µmol m^−2^ s^−1^) and high sucrose (c.a. 3%). In general (Fig. 1E), sucrose favored the production of new *ex vitro* leaves; whereas mid and high light intensities inhibited their development during *ex vitro* rooting and acclimation. A 3-D plotting of the relationship between the number of *ex vitro/in vitro* leaves rate (Fig. 1F) revealed that the lowest ratio was found at a low sucrose concentration and high light intensity level.

The 3D plot predicting plantlet dry weight (Fig. 1G) showed a complex non-linear interaction between the inputs. As it has been shown for other growth parameters, such as root length (Fig. 1B), shoot length (Fig. 1C) and plantlet water content (WC) percentage (Fig. 1H), increments in light intensity up to approx. 122 µmol m^−2^ s^−1^ resulted in an increase of plantlet dry weight. The highest light intensities however reduced the plantlet dry weight. Consequently, the highest dry weights were obtained at a high sucrose level (c.a. 2.33%) combined with mid (in the range of 122–138 µmol m^−2^ s^−1^) light intensities (Fig. 1G).

A 3D plot predicting the WC (Fig. 1H) indicated that sucrose increased the WC percentage at all light intensities. Light intensity, however, promotes higher WC values only until a threshold is reached (in the range of 122–138 µmol m^−2^ s^−1^), since WC decreased significantly with higher irradiances. In conclusion, the most elevated WC values were obtained at maximal sucrose levels plus mid light intensities (Fig. 1H). This pattern is in line with that observed for other growth parameters such as root length, shoot length and dry weight (Fig. 1B, C, G). The multiscale analysis created a clear display of the complex effects and interactions between sucrose and light on plantlet growth. Sucrose supplementation appeared to be essential in order to reach the optimal values of most of the growth parameters studied (at 2.3% or higher concentration) independent or in interaction with mid light intensities (122–138 µmol m^−2^ s^−1^).

### Tissue Scale

**Figure 2:**
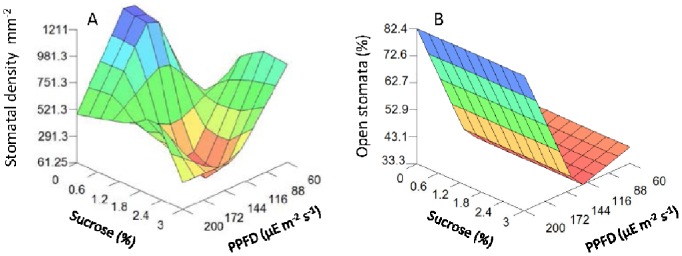
3D plots of chlorophyll fluorescence parameters predicted by the neurofuzzy logic model for kiwifruit plantlets at 42 d at acclimatization stage as a function of sucrose added in the medium and light intensities used during *in vitro* culture. (A) F_v_/F_m_. (B) F_0._

The stomatal density differed considerably among treatments, ranging mostly from 300 to 900 stomata per mm^2^ (Table 2), with no abnormal stomata found. The model revealed a significant complex non-linear interaction between light and sucrose on stomatal density (Table 3; Fig. 2). The lowest stomatal density (Fig. 2A) was achieved using a sucrose concentration of approximately 2–2.3% and medium light intensities (in the range of 122–138 µmol m^−2^ s^−1^) and the highest stomatal density observed when the concentration of sucrose was nearly 0% at the same intensity of light.

Light intensity also had a significant effect on the number of open stomata output (Fig. 2B). The highest percentage values of open stomata were found at high light intensity (c.a. 200 µmol m^−2^ s^−1^), and the lowest again at mid level light intensity (around 122 µmol m^−2^ s^−1^) (Fig. 2B). Low light levels (c.a. 60 µmol m^−2^ s^−1^) also resulted in a higher percentage of open stomata compared to the mid level light intensity. Finally, the proportion of open stomata was not dependent on the sucrose concentration at any given light intensity (Fig. 2B).

### Subcellular Scale

**Figure 3:**
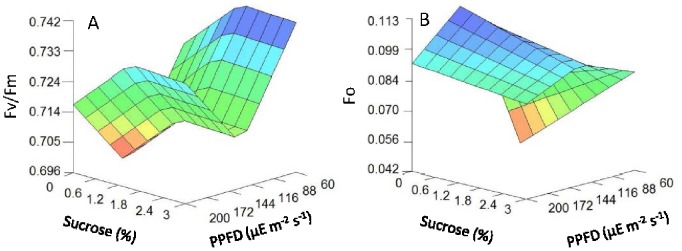
3D plots of stomatal parameters predicted by the neurofuzzy logic model for kiwifruit plantlets at 45 d at acclimatization stage as a function of sucrose added in the medium and light intensities used during *in vitro* culture. (A) Stomatal density (mm^−2^). (B) Proportion of open stomata (%).

The model predicts that light had the strongest effect on the photosynthetic quantum efficiency of PSII (F_v_/F_m_) (Table 3; Fig. 3A), which reached the minimum value when plantlets were grown under medium light intensity (122–138 µmol m^−2^ s^−1^). Sucrose had an independent and secondary effect on photosynthetic quantum efficiency of PSII, which achieved maximum values when sucrose concentrations were at a medium level (approx. 1.6%). Therefore, the maximum value of F_v_/F_m_ (0.7428) was achieved at low light intensities (60 µmol m^−2^ s^−1^) and medium sucrose concentrations (around 1.6%); while the minimum value (0.70) was reached at a very low sucrose concentration (lower than 1.6%) and medium light intensity.

F_v_/F_m_ predictions agreed with F_0_ estimated values (Fig. 3B), as seen with the higher basal fluorescence F_0_ values found under medium light treatments (in the range of 122–138 µmol m^−2^ s^−1^) and at very low sucrose concentrations (c.a. 0.01%).

**Figure 4:**
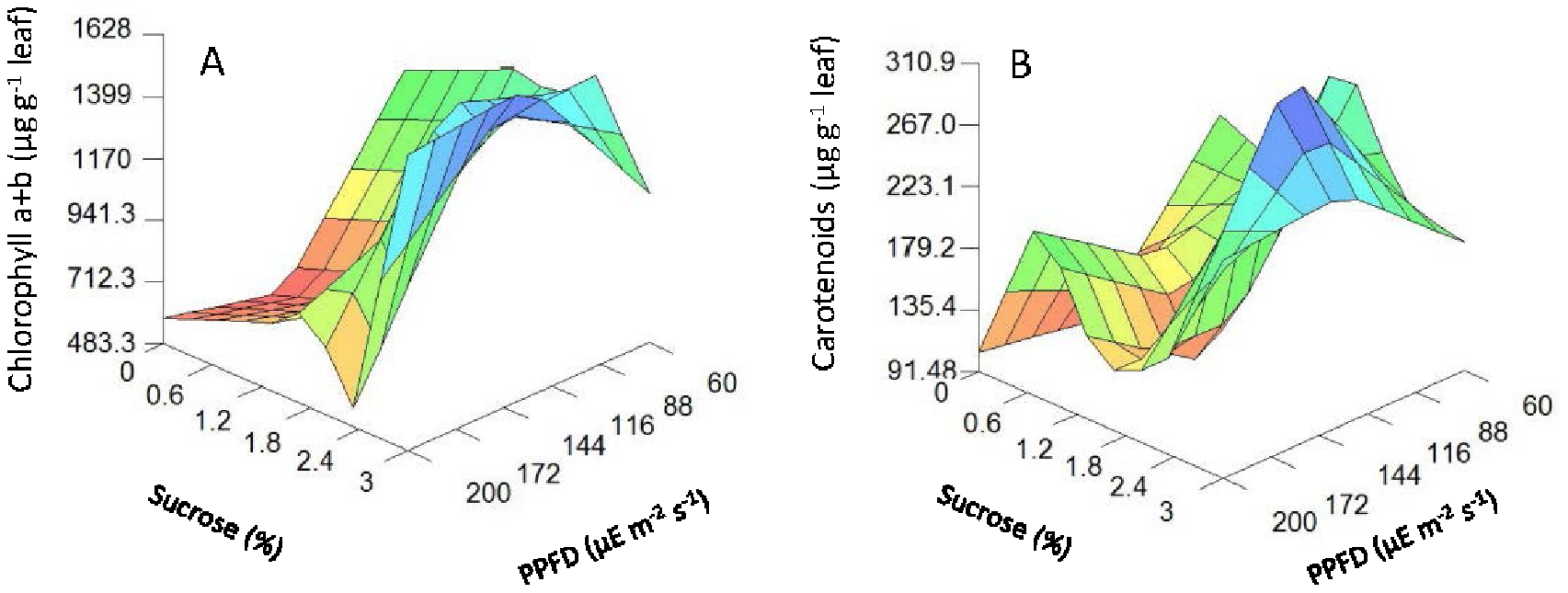
3D plots of photosynthetic pigment parameters predicted by the neurofuzzy logic model for kiwifruit plantlets at 45 d at acclimatization stage as a function of sucrose added in the medium during *in vitro* culture and light intensities used. (A) chlorophyll a+b (µg g^−1^ leaf). (B) carotenoids (µg g^−1^ leaf) content.

The model also indicated a significant complex non-linear interaction between light intensity and sucrose concentration on the photosynthetic pigment contents (Table 3 and Fig. 4). High sucrose concentrations (up to 2.3%) and mid light intensities (in the range of 122–138 µmol m^−2^ s^−1^) were required to promote the highest content in total chlorophyll a+b content (Fig. 4A), whereas mid sucrose concentration (2.3%) and mid light intensity (122 µmol m^−2^ s^−1^) were required for the highest carotenoid content (Fig. 4B). These results pointed towards light intensity being the determinate variable in chlorophyll fluorescence and stomatal open parameters, regardless of sucrose concentration.

In this study, fourteen sets of “IF - THEN” rules were extracted, from the submodels, for each one of the outputs studied (see supplementary data; Table S1). As an example, Table 4 presents the set of rules for some combinations that produced the highest membership value for each output (>0.83). By interpreting the rules in Table 4, useful relationships can be observed, for example in rule 1: IF the sucrose concentration in the medium is low THEN the percentage of survival is almost always low (membership degree 0.90) regardless of the light level used (Table 4). Similarly, IF the sucrose concentration is high THEN root length, shoot length, number of *ex vitro* leaves, and WC are definitely high (Table 4; Submodel 1; Rules 2, 4, 7 and 12; membership degree 1.00). Therefore, a low sucrose concentration significantly reduces these parameters (Table S1). On the contrary, light is clearly the most important factor for open stomata and the photosynthetic (F_v_/F_m_) parameters (Table 4; rules 19; 20–21). Interestingly, IF mid light intensity is applied THEN low F_v_/F_m_ (rule 21; 1.00 membership) and a low percentage of open stomata (rule 19; 0.83 membership) are predicted; low F_v_/F_m_ and percentages of open stomata are modeled independently of sucrose concentration.

**Table 4:** Most relevant rules with higher memberships generated by neurofuzzy logic software for each output.

Rule	Submodel		Sucrose (%)	LightIntensity			Output	*Membership degree*
1	1	**IF**	Low	–	**THEN**	Low	Survival (%)	0.90
2	1		High	–		High	Root length (cm)	1.00
3	2		–	High		Low		1.00
4	1		High	–		High	Shoot length (cm)	1.00
5	2		–	High		Low		0.93
6	1		Mid 2(4)	High		High	*In vitro* leaves per plantlet	1.00
7	1		High	–		High	*Ex vitro* leaves per plantlet	1.00
8	2		-	Low		High		0.92
9	1		Low	High		Low	*Ex vitro/in vitro* leaves	0.99
10	1		Mid 4(5)	Mid		High	Plantlet dry weight (g)	1.00
11			Mid 2(5)	High		Low		1.00
12	1		High	–		High	WC (%)	1.00
13	2		–	High		Low		0.92
14	1		Mid 2(5)	Low		Low	Stomatal density (mm^2^)	1.00
15			Mid 1(5)	Mid		High		1.00
16			Mid 2(5)	Mid		High		1.00
17			Mid 3(5)	Mid		Low		1.00
18			Mid 4(5)	Mid		Low		1.00
19	1		–	Mid		Low	Open Stomata (%)	0.83
20	1		–	Low		High	F_v_/F_m_	1.00
21			–	Mid		Low		1.00
22	2		Low	–		Low		1.00
23	1		Low	Low		Low	F_0_	1.00
24			Low	Mid		High		1.00
25			High	Mid		High		1.00
26	1		Low 1(5)	Mid		Low	Chl a+b (µg g^−1^ leaf)	1.00
27			Mid 4(5)	High		Low		1.00
28			High 5(5)	Mid		High		1.00
29			High 5(5)	High		High		1.00
30	1		Low 1(5)	Mid		Low	Carotenoids (µg g^−1^ leaf)	1.00
31			Low 1(5)	High		Low		1.00
32			Mid 3(5)	Mid		Low		1.00
33			Mid 4(5)	High		Low		1.00

Both variables, sucrose and light, have significant interactions affecting the remainder of the parameters studied (Table 4): number of *in vitro* leaves (rule 6), *ex vitro/in vitro* leaf ratio (rule 9), plantlet dry weight (rules 10–11), stomatal density (rules 14–18), F_0_ (rules 23–25), chlorophyll a+b (rules 26–29) and carotenoid content (rules 30–33).

## Discussion

Biological processes are both time variant and non-linear in nature, and their complexity can be understood as the composition of many different and interacting elements governed by non-deterministic rules and influenced by external factors [50], [65]. Taking this into account, researchers cannot expect to obtain a full understanding of plant processes by focusing on only one level of organization [39], such as growth parameters at whole plant level. Indeed, recent reviews have pointed out the importance of integrating the different scales of biological organization from different levels of organization to shift the typical “reductionist view” towards a “holistic” view, to reach a more realistic, yet also more complex context [59], [66]–[67]. The complexity of plant responses and interactions between biological scales must be taken into account to determine, or predict, with greater accuracy what is happening in plants at any scale, stage or condition and to obtain a more real understanding of the processes involved at the whole-plant scale [47], [67]–[68].

Artificial intelligence techniques can be used as new and powerful tools for navigating different levels of complexity, and modeling complex non-linear relationships concealed within datasets [43]–[44], [51], [55]–[56]. To our knowledge, and according to recent reviews of plant systems biology and functional modeling [49], [59], [66], there are no previous reports describing the use of models derived by using artificial intelligence methods to integrate and model complex multi-scale datasets in plant science. Acclimation of *in vitro* propagated plants to *ex vitro* conditions still remains poorly understood [5] and entails an understanding of the effects of *in vitro* culture conditions upon several parameters at different biological levels.

The neurofuzzy logic approach not only identified the significant effect of sucrose on the main growth parameters usually employed as references of plantlet acclimation and quality: survival, roots and shoot length, *ex vitro* leaves per plantlet and WC, which was clearly independent of light regimes; but also showed that light plays a significant effect on only two parameters directly related to photoautotrophy and photoinhibition i.e. F_v_/F_m_ at the subcellular level and the proportion of open stomata at the tissue level, without interaction with the sucrose concentration. However, at the mid light intensities promoting the highest growth, these two parameters were lower in comparison to the other treatments. For instance, maximum F_v_/F_m_ was about 0.75, in coincidence with typical values observed in *in vitro* plants [31]–[32], [69], and was slightly but significantly lower at the mid light intensity conditions than at low or somewhat higher intensities, suggesting that the lowest rates of photosynthesis occurred at mid light. These low photosynthetic values were accompanied by the highest F_0_ values, which is an indicator of chronic photoinhibition or photoinactivation [70]–[72]. Using the equation proposed by Evans and Poorter [73] to estimate leaf absorptance from chlorophyll content as α = [Chl]/([Chl]+76), the linear electron transport rate (ETR) from ETR = φ_e_×PAR×0.5×α. ETR values were ca. 40 µmol e m^−2^ s^−1^ for low light plants, and were estimated for grown plants at around 30 µmol e m^−2^ s^−1^ for mid and high light intensity. Therefore, at the mid light intensities, promoting the best plant growth, photosynthesis may be similar or even somewhat lower than at other light intensities, indicating that plant growth is not strongly related to photosynthesis rate under these conditions. Despite similar or even lower photosynthesis rate at mid light, photosynthetic pigment content (chlorophylls and carotenoids) was the highest at these intensities, supporting previous results described elsewhere in other materials [31]–[32]. However, the effect was greater for carotenoids, as the ratio Car/Chl increased, suggesting that plants were in a more photoprotective stage [74]. Finally, at mid light intensities and high sucrose content, both stomatal density per mm^2^ and the percentage of open stomata were at their lowest values, indicating that stomatal conductance was also at its lowest value. Previous reports on different species have suggested that restricted stomatal openness is a more limiting factor for photosynthesis of *in vitro* cultured plants than light intensity [6], [31], [75]. In fact, upon transfer to *ex vitro* conditions, progressive stomatal closure has been described as a key response of plants [11], [33], [69], [76]. Since stomatal closure has the penalty of reduced CO_2_ diffusion and, hence photosynthesis [40], this may explain the need for a higher photoprotective state of mid light plants. However, closed stomata prevent water loss, which has also been described, together with excessive light, as one of the major problems for enduring *in vitro* plants when transferred to *ex vitro* conditions [19]–[21]. Indeed, the present results show that plant WC was the highest for mid light grown plants, coinciding with the lowest area of stomatal aperture and the highest plant growth (here measured as root and shoot length and dry weight). It is well established that cell turgor associated with high WC is essential for plant cell enlargement, which may at least in part explain these observations.

## Conclusions

Here we demonstrate that Artificial Intelligence can be useful as one of the key technologies in modeling complex plant systems, and that it is capable, (specifically neurofuzzy logic techniques), of deriving useful, valuable knowledge, using a holistic scope. Although we have used kiwifruit plants, this technology can be applied to any other plant species.

Through a neural fuzzy approach we have been able to discover new complex interactions among the inputs studied and the consequences of varying both sucrose concentration and light intensity (0 to 3% sucrose and 60 to 200 µmol m^−2^ s^−1^ PPFD) in *in vitro* kiwifruit microshoots to its acclimation. In fact, the present results are a clear illustration that, at least under the light-limited environment used in the present study, the most critical threat for *in vitro* cultured kiwi plantlets following transfer to *ex vitro* conditions is water availability/balance rather than excess light intensity. This provides an explanation suggesting that plants showing the lowest area of stomatal aperture, the highest level of photoinhibition and photoprotective responses are better prepared for acclimatization to *ex vitro* because they maintain the appropriate water status. Finally, the use of a neurofuzzy logic technology allowed us to deduce the best plant growth conditions (2.3% sucrose and 122–130 µmol m^−2^ s^−1^), taking in account all the parameters measured, required for the highest quality of acclimatized plantlets to be obtained and increased our understanding of the interactions and the role of the main factors involved in plant acclimation.

## Supporting Information

Table S1 **Rules set for each output generated by neurofuzzy logic.**
(DOC)
