# Supplementary material for: Modeling the Effects of Light and Sucrose on In Vitro Propagated Plants: A Multiscale System Analysis Using Artificial Intelligence Technology
Source: PLoS One. 2014 Jan 20;9(1):e85989. doi: 10.1371/journal.pone.0085989 (PMC3896442; doi:10.1371/journal.pone.0085989)
Supplement: Table S1 — Rules set for each output generated by neurofuzzy logic. (DOC) [file pone.0085989.s001.doc]

Supplementary data:

**Modeling the effects of light and sucrose on in vitro propagated plants: a multiscale system analysis using artificial intelligence technology**

**.**

**J. Gago ^a^, L. Martínez-Núñez ^a^, M. Landín ^b^, J. Flexas ^c^, P.P. Gallego ^a,*^.**

Table S1. Rules set for each output generated by neurofuzzy logic.

| Submodel |  | Sucrose (%) | Light Intensity |  |  | Output | *Membership degree* |
| --- | --- | --- | --- | --- | --- | --- | --- |
| 1 | IF | Low | - | THEN | Low | Survival (%) | 0.90 |
|  |  | Mid | - |  | High |  | 0.84 |
|  |  | High | - |  | High |  | 0.84 |
| 1 | IF | Low | - | THEN | Low | Root length (cm) | 0.91 |
|  |  | High | - |  | High |  | 1.00 |
| 2 |  | - | Low |  | Low |  | 0.60 |
|  |  | - | Mid |  | High |  | 0.98 |
|  |  | - | High |  | Low |  | 1.00 |
| 1 | IF | Low | - | THEN | Low | Shoot length (cm) | 0.99 |
|  |  | High | - |  | High |  | 1.00 |
| 2 |  | - | Low |  | Low |  | 0.58 |
|  |  | - | Mid |  | High |  | 0.76 |
|  |  | - | High |  | Low |  | 0.93 |
| 1 | IF | Low_1(4) | Low | THEN | Low | *In vitro* leaves | 0.63 |
|  |  | Low_1(4) | High |  | High |  | 0.95 |
|  |  | Mid_2(4) | Low |  | Low |  | 0.69 |
|  |  | Mid_2(4) | High |  | High |  | 1.00 |
|  |  | Mid_3(4) | Low |  | Low |  | 0.53 |
|  |  | Mid_3(4) | High |  | Low |  | 0.81 |
|  |  | High_4(4) | Low |  | Low |  | 0.70 |
|  |  | High_4(4) | High |  | Low |  | 0.91 |
| 1 | IF | Low | - | THEN | Low | *Ex vitro* leaves | 0.86 |
|  |  | Mid | - |  | Low |  | 0.96 |
|  |  | High | - |  | High |  | 1.00 |
| 2 |  | - | Low |  | High |  | 0.92 |
|  |  | - | Mid |  | Low |  | 0.82 |
|  |  | - | High |  | Low |  | 0.84 |
| 1 | IF | Low | Low | THEN | High | *Ex vitro/in vitro* leaves | 0.52 |
|  |  | Low | Mid |  | Low |  | 0.94 |
|  |  | Low | High |  | Low |  | 0.99 |
|  |  | High | Low |  | High |  | 0.74 |
|  |  | High | Mid |  | High |  | 0.66 |
|  |  | High | High |  | High |  | 0.95 |
| 1 | IF | Low | Low_1(5) | THEN | Low | Dry weight (g) | 0.64 |
|  |  | Low | Mid_2(5) |  | High |  | 0.56 |
|  |  | Low | Mid_3(5) |  | High |  | 0.62 |
|  |  | Low | Mid_4(5) |  | High |  | 0.74 |
|  |  | Low | High_5(5) |  | High |  | 0.52 |
|  |  | Mid | Low_1(5) |  | Low |  | 0.67 |
|  |  | Mid | Mid_2(5) |  | High |  | 0.93 |
|  |  | Mid | Mid_3(5) |  | High |  | 0.81 |
|  |  | Mid | Mid_4(5) |  | High |  | 1.00 |
|  |  | Mid | High_5(5) |  | High |  | 0.67 |
|  |  | High | Low_1(5) |  | Low |  | 0.72 |
|  |  | High | Mid_2(5) |  | Low |  | 1.00 |
|  |  | High | Mid_3(5) |  | Low |  | 0.68 |
|  |  | High | Mid_4(5) |  | High |  | 0.77 |
|  |  | High | High_5(5) |  | High |  | 0.57 |
| 1 | IF | Low | - | THEN | Low | WC (%) | 0.93 |
|  |  | High | - |  | High |  | 1.00 |
| 2 |  | - | Low |  | Low |  | 0.65 |
|  |  | - | Mid |  | High |  | 0.88 |
|  |  | - | High |  | Low |  | 0.92 |
| 1 | IF | Low | Low_1(5) | THEN | Low | Stomatal density  mm^-2^ | 0.91 |
|  |  | Low | Mid_2(5) |  | Low |  | 1.00 |
|  |  | Low | Mid_3(5) |  | High |  | 0.74 |
|  |  | Low | Mid_4(5) |  | High |  | 0.52 |
|  |  | Low | High_5(5) |  | High |  | 0.95 |
|  |  | Mid | Low_1(5) |  | High |  | 1.00 |
|  |  | Mid | Mid_2(5) |  | High |  | 1.00 |
|  |  | Mid | Mid_3(5) |  | Low |  | 1.00 |
|  |  | Mid | Mid_4(5) |  | Low |  | 1.00 |
|  |  | Mid | High_5(5) |  | Low |  | 0.96 |
|  |  | High | Low_1(5) |  | Low |  | 0.67 |
|  |  | High | Mid_2(5) |  | Low |  | 0.59 |
|  |  | High | Mid_3(5) |  | High |  | 0.50 |
|  |  | High | Mid_4(5) |  | High |  | 0.70 |
|  |  | High | High_5(5) |  | Low |  | 0.94 |
| 1 | IF | - | Low | THEN | Low | Open stomata (%) | 0.75 |
|  |  | - | Mid |  | Low |  | 0.83 |
|  |  | - | High |  | High |  | 0.78 |
| 1 | IF | - | Low | THEN | High | F_v_/F_m_ | 1.00 |
|  |  | - | Mid |  | Low |  | 1.00 |
|  |  | - | High |  | High |  | 0.65 |
| 2 |  | Low | - |  | Low |  | 1.00 |
|  |  | Mid | - |  | High |  | 0.70 |
|  |  | High | - |  | High |  | 0.66 |
| 1 | IF | Low | Low | THEN | Low | F_0_ | 1.00 |
|  |  | Low | High |  | High |  | 0.86 |
|  |  | Mid | Low |  | High |  | 1.00 |
|  |  | Mid | High |  | High |  | 1.00 |
|  |  | High | Low |  | High |  | 0.95 |
|  |  | High | High |  | High |  | 0.83 |
| 1 | IF | Low_1(5) | Low | THEN | High | Chl a+b  (µg g^-1^ leaf) | 0.53 |
|  |  | Low_1(5) | Mid |  | Low |  | 1.00 |
|  |  | Low_1(5) | High |  | Low |  | 0.97 |
|  |  | Mid_2(5) | Low |  | High |  | 0.72 |
|  |  | Mid_2(5) | Mid |  | Low |  | 0.92 |
|  |  | Mid_2(5) | High |  | Low |  | 0.80 |
|  |  | Mid_3(5) | Low |  | High |  | 0.51 |
|  |  | Mid_3(5) | Mid |  | High |  | 0.66 |
|  |  | Mid_3(5) | High |  | Low |  | 0.92 |
|  |  | Mid_4(5) | Low |  | High |  | 0.61 |
|  |  | Mid_4(5) | Mid |  | Low |  | 0.78 |
|  |  | Mid_4(5) | High |  | Low |  | 1.00 |
|  |  | High_5(5) | Low |  | Low |  | 0.53 |
|  |  | High_5(5) | Mid |  | High |  | 1.00 |
|  |  | High_5(5) | High |  | High |  | 1.00 |
| 1 | IF | Low_1(5) | Low | THEN | High | Carotenoids  (µg g^-1^ leaf) | 0.51 |
|  |  | Low_1(5) | Mid |  | Low |  | 1.00 |
|  |  | Low_1(5) | High |  | Low |  | 1.00 |
|  |  | Mid_2(5) | Low |  | Low |  | 0.60 |
|  |  | Mid_2(5) | Mid |  | Low |  | 0.71 |
|  |  | Mid_2(5) | High |  | High |  | 0.65 |
|  |  | Mid_3(5) | Low |  | High |  | 098 |
|  |  | Mid_3(5) | Mid |  | Low |  | 1.00 |
|  |  | Mid_3(5) | High |  | Low |  | 0.86 |
|  |  | Mid_4(5) | Low |  | Low |  | 0.73 |
|  |  | Mid_4(5) | Mid |  | High |  | 0.86 |
|  |  | Mid_4(5) | High |  | Low |  | 1.00 |
|  |  | High_5(5) | Low |  | Low |  | 0.57 |
|  |  | High_5(5) | Mid |  | High |  | 0.84 |
|  |  | High_5(5) | High |  | High |  | 0.79 |
